# Psychometric evaluation of a new love scale, measuring the content and modality dimensions, based on love and rejection messages theory

**DOI:** 10.3389/fpsyg.2026.1806481

**Published:** 2026-08-14

**Authors:** Ioana-Eva Cǎdariu, Loredana-Ileana Vîşcu, Marius Costel Eşi, Marius Marici

**Affiliations:** 1 Institute of Psychotherapy, Psychological Counseling and Clinical Supervision, Tibiscus University of Timişoar, Timişoara, Romania; 2 Faculty of Educational Sciences, Stefan cel Mare University, Suceava, Romania

**Keywords:** construct validity, factor structure, incremental validity, love assessment, LRMT, psychometric validation, reliability

## Abstract

**Introduction:**

Existing measures of romantic love primarily assess global feelings, attitudes, or relationship satisfaction, providing limited insight into how love is communicated in everyday interactions.

**Methods:**

The present study evaluated the psychometric properties of the Marici Love Messages Scale (MLMS), developed within the Love and Rejection Messages Theory (LRMT) to assess two complementary dimensions of love messages: Content (what partners do) and Modality (how these actions are expressed). Two independent community samples were used for exploratory and confirmatory analyses.

**Results:**

The findings supported the proposed factor structure and demonstrated high internal consistency for both subscales. Strong associations with Sternberg's dimensions of intimacy, passion, and commitment provided evidence of convergent validity, while discriminant validity was also supported. Measurement invariance across gender was supported at the configural, metric, scalar, and strict levels. Regression analyses further indicated that the Content and Modality dimensions explained unique variance beyond Sternberg's model, supporting their incremental validity.

**Discussion:**

The results suggest that the MLMS is a reliable and valid instrument for assessing everyday expressions of love and may be useful in both relationship research and6 clinical practice.

## 1 Theoretical Introduction

Love was considered for a period a topic which is not subject to scientific inquiry as it was viewed too subjective, private and culturally saturated (Berscheid and Hatfield, 1969). Yet, science succeeded to develop measures to capture the complexity of romantic love, at present there are several dozen measurement instruments.

### 1.1 Measures of love construct

Until now, romantic love was measured with a variety of widely used psychometric instruments. Among these, the Passionate Love Scale developed by Hatfield and Sprecher operationalizes passionate love through cognitive, emotional, and behavioral indicators (Hatfield and Sprecher, 1986); Rubin's Love Scale distinguishes love from liking via attachment, caring, and intimacy (Rubin, 1970); Sternberg's Triangular Love Scale measures intimacy, passion, and commitment components (Sternberg, 1988, 1997); and the Love Attitudes Scale captures multiple love styles derived from Lee's color wheel model (Hendrick and Hendrick, 1986). DAS (Dyadic Adjustment Scale) (Spanier, 1976) measures relationship satisfaction, cohesion, consensus and affectional expression.

At the same time, most existing instruments operate at a relatively global level of analysis—capturing how much love is experienced, which attitudes or styles define it, or how satisfied partners are overall (Marici, 2025a). While this global assessment is highly valuable, it may be less informative when the aim is to understand the everyday interpersonal mechanisms through which love is maintained, communicated, disrupted, and restored.

### 1.2 The need for a new scale

LRMT is a recent theory that claims that love can be defined as perceptions derived from actions done in a caring way, which are part of fluxes of love messages, which circulate between partners every day. As informed from clinical practice, these love messages are interactional, perceptual and subjective. Partners often report performing actions that should convey love. For example, giving gifts, helping or spending time together. Yet these actions may not be experienced as messages of love. Instead, they may be perceived as routine, conditional, mismatched, delayed, or emotionally disconnected, sometimes even as rejection. This shows that relational meaning is not defined only by the existence of an action, but also by the modality/modalities of its delivery (e.g., timing, initiative, spontaneity, empathy, attunement to preferences, or the priority given to the relationship). Identical behaviors can generate opposite emotional consequences, because of different modalities.

The present study is the first to validate a measure based on the two components of love messages as described in the Love and Rejection Messages Theory (LRMT). The motivation to develop a new measure was fueled by the perspective that a clinically based instrument may be more promising in measuring and treating lack of romantic love better, by focusing of the flux of love messages. LRMT was chosen as the basis because it is a new theoretical framework that views love as a dynamic flow of love messages between partners. A lack of an optimal flow of love messages reflects an insufficient or dysfunctional romantic relationship, as partners need to perceive that they are loved and to express love every day. Measuring the present exchange of love messages, rather than the overall perception of love, may be more valuable because it can inform specialists about exactly what needs to be improved and even how. Existing scales, as they were not developed within this theoretical framework, cannot measure the constructs proposed by the theory. What is more this scale can be an indicator of therapeutic change in a pre-post intervention analysis.

### 1.3 Love and rejection messages theory

The theory proposes that couple functioning is shaped by the interpersonal flux of messages that are experienced as either love or rejection. Romantic love is not treated solely as an internal feeling or global evaluation. It is rather as an emergent relational phenomenon, sustained through repeated exchanges between partners. The possibility of “rekindling” love depends on two complementary processes: increasing the flux (circulation) of love messages and effectively managing the flux (circulation) of rejection messages.

Love messages are composed of two interrelated components. *Content of love messages*, referring to the actions themselves, what the partner does in everyday life to convey love. They may refer to listening, spending time together, respecting privacy, expressing affection, supporting autonomy, engaging sexually etc. The second is modality of love messages. They refer to how these actions are delivered by one partner and experienced by the other. Whether the behavior is spontaneous rather than calculated, initiated freely rather than under pressure, emotionally attuned, empathic, timely, and aligned with the partner's preferences and meanings, etc. Content without appropriate modality may fail to function as a love message. Modality can transform ordinary actions into powerful signals of being valued and prioritized. When content is absent or modality is incongruent, partners may experience the interaction as rejection, even if no overt rejection is intended.

On this basis, the Marici Love Messages Scale (MLMS) was developed to assess the two LRMT domains: *Content of Love Messages* and *Modality of Love Messages*, as theoretically connected dimensions of couple functioning.

## 2 Method

### 2.1 Aim of the present research

The aim of the present research is to explore the psychometric properties of a new love scale, made up of two dimensions: content of love messages and modality of love messages.

### 2.2 Participants

The sample consisted of 509 participants (54.6% women). Participants had a mean age of 30.87 years (*SD* = 10.70; range = 18–64) and had been in their current romantic relationship for an average of 9.25 years (*SD* = 8.97; range = 0–40). Most participants lived with their partner in their own home (78.6%), had no children (58.0%), held a bachelor's (45.8%) or master's degree (35.2%), and reported an average family financial situation (70.5%) (see Table 1).

**Table 1:** Characteristics of participants.

Variable	Category/statistic	*n*	%
Gender	Female	278	54.6
	Male	231	45.4
Age (years)	*M* = 30.87, *SD* = 10.70, Range = 18–64		
Relationship duration (years)	*M* = 9.25, *SD* = 8.97, Range = 0–40		
Living arrangement	Living with his parents	38	7.5
	Living with my parents	71	13.9
	Living together in own home	400	78.6
Number of children	None	295	58.0
	One	100	19.6
	Two	73	14.3
	Three	28	5.5
	Four or more	13	2.6
Education level	Secondary education	20	3.9
	Bachelor's degree	233	45.8
	Master's degree	179	35.2
	PhD	74	14.5
	Other	3	0.6
Family financial situation	Very low	3	0.6
	Low	40	7.9
	Average	359	70.5
	Good	91	17.9
	Very good	16	3.1

The first sample (*N* = 509) was used for the exploratory factor analyses and the subsequent reliability and construct validity analyses. An independent second sample was used for the confirmatory factor analyses to cross-validate the proposed measurement models. The second sample consisted of 339 individuals. Participants ranged in age from 18 to 75 years (*M* = 36.43, SD = 11.53). The average relationship duration was 13.10 years (SD = 10.60). Women represented 56.3% of the sample, and nearly three quarters of participants were married (73.5%). Most participants lived with their partner in their own home (83.2%) and had at least a secondary education. About two thirds rated their family's financial situation as average. Only 2.9% of participants reported previous experience with couple therapy. The sample were recruited from the general population and reflected predominantly non-clinical populations.

Differences in sample size across analyses were due to missing data and listwise deletion.

Participants were recruited through convenience sampling using an online survey. The sample size was determined based on established recommendations for factor analysis (at least 10 participants per item), and the final sample exceeded this criterion.

### 2.3 Measures

The present study used the following instruments. They represent clinically meaningful expressions of relational distress, found in couple therapy.

#### 2.3.1 The Sternberg triangular love scale

Sternberg, 1988, 1997 is a self-report instrument designed to measure romantic love according to the Triangular Theory of Love. It assesses three dimensions: Intimacy, Passion, and Commitment. The original scale contains 45 items. There are 15 items per dimension. Each item is rated on a Likert-type scale (1 = not at all to 9 = extremely). Higher scores indicate greater levels of love. The scale has been widely validated across cultures, showing strong reliability and construct validity.

#### 2.3.2 Desire for physical separation (DPS)

The instrument (Marici, 2025b) measures the subjective desire to increase physical distance from one's romantic partner. The scale consists of 6 items assessing avoidance-oriented tendencies, the need for space, and the emotional relief associated with being physically apart from the partner. Responses are dichotomous, yes/no, with higher scores indicating a stronger desire for physical separation. Alpha Cronbach for the scale is 0.733.

#### 2.3.3 Dysfunctional communication (DC)

Assesses the problematic communication (Marici, 2025b). The scale is designed to capture the extent to which everyday communication prevents optimum couple functioning. The instrument consists of 6 items. Responses are recorded on a 6-point Likert scale ranging from 0 (“Totally disagree”) to 5 (“Totally agree”). Higher scores indicate dysfunctional communication. An example item is: “*I cannot have an honest conversation with my partner about us because they do not understand*.”

#### 2.3.4 Emotional Infidelity (EI)

The Emotional Infidelity (EI) instrument (Marici, 2025b) measures the degree to which significant emotional investment is redirected outside the couple relationship, without referring explicitly to sexual behavior. The instrument includes 5 unidimensional items, and responses are dichotomous, yes/no, the total scores ranging from 0 to 25. Higher scores reflect a greater level of Emotional Infidelity. An example item is: “*Unlike with my partner, I can talk about anything, even personal problems, with this person*.”

#### 2.3.5 Negative Perception of Sex Life (NPOSL)

Instrument (Marici, 2025b) evaluates the negative cognitive–emotional perception of the couple's sex life. The scale does not assess sexual frequency or performance, but rather the subjective meaning attributed to sexual experiences within the relationship. The instrument comprises 7 items, with responses rated on a 6-point Likert scale ranging from 0 (“Totally disagree”) to 5 (“Totally agree”). Higher scores indicate a more negative perception of the sexual relationship. An example item is: “*I don't feel truly desired by my partner*.” Alpha Cronbach for the scale is 0.852.

#### 2.3.6 Hope in the relationship with the Partner (HRP)

The measure assesses the level of relational hope, defined as the psychological availability to invest in repairing and continuing the relationship despite difficulties (Marici, 2025b). The instrument consists of 7 items. The responses are rated on a 6-point Likert scale, ranging from 0 (“Totally disagree”) to 5 (“Totally agree”). Higher scores reflect greater relational hope, which functions as a key protective factor against the desire for physical/emotional separation. An example item is: “*I believe I will have a happy life with my partner moving forward*.” The total score is formed by adding all individual scores. The total scale scores can be between 0 and 35. Alpha Cronbach for the scale is 0.899.

#### 2.3.7 Marici Love Messages Scale (MLMS)

The scale was created by Marici Marius on Love and Rejection Messages Theory (Marici, 2025a,b), consisting of two theorized dimensions: Content of Love Messages (CLM), and Modality of Love Messages (MLM). Content refers to the actions themselves, while modality refer to the way actions are done.

Initially the items were observed during therapeutic sessions and retained, and later a group of experts decided which items from the total pool of items are the most important and which not. Content validity was established through expert review. Three psychologists with expertise in couple and family psychology evaluated each item for clarity, relevance, representativeness, and consistency with the theoretical definitions of the Content and Modality dimensions. Based on their feedback, items were refined to improve conceptual accuracy and alignment with the Love and Rejection Messages Theory.

Before the psychometric analyses, the items were refined through repeated clinical discussions with couples during the development and application of the Love and Rejection Messages Theory. This process helped ensure that the items were clear, meaningful, and consistent with the experiences they were intended to capture.

For CLM scale, respondents were asked to indicate, according to their perception, how often their partner does those actions, as part of couple life in the last 6 months, with them. The answers were indicated on a 3-point scale: 1 (absent sau puţin/absent or little), 2 (insuficient/insufficient), 3 (suficient sau mai mult/enough or much more). Alpha Cronbach for the CLM scale with 14 items was 0.906 and for the MLM scale, with 14 items, was 0.964.

### 2.4 Statistical analysis strategy

The analyses were conducted to evaluate the factorial structure, reliability, and construct validity of the LRMT-based Love Scale and to test its added value beyond Sternberg's Triangular Theory of Love. The later scale was chosen as it is a reliable scale with psychometric properties in most cultures (Sorokowski et al., 2021).

Some statistical analyses were performed in R (version 4.4; R Core Team, 2024). Exploratory factor analyses (EFA) were conducted using the psych package (Revelle, 2023) with maximum likelihood extraction and Oblimin rotation in r or in Jamovi. Confirmatory factor analyses (CFA) were performed using the lavaan package (Rosseel, 2012), applying the WLSMV estimator for ordinal data and the MLR estimator when appropriate. Model fit was evaluated using the χ^2^ statistic, Comparative Fit Index (CFI), Tucker–Lewis Index (TLI), Root Mean Square Error of Approximation (RMSEA) with 90% confidence intervals, and the Standardized Root Mean Square Residual (SRMR).

Internal consistency was assessed using Cronbach's alpha and McDonald's omega with the psych package. Convergent validity was examined using Pearson correlation analyses, whereas discriminant validity was evaluated using Composite Reliability (CR), Average Variance Extracted (AVE), Maximum Shared Variance (MSV), and the Fornell–Larcker criterion, computed with the semTools package (Jorgensen et al., 2022). Criterion-related and incremental validity were assessed using hierarchical multiple regression analyses. The additional predictive value of the Content and Modality dimensions beyond Sternberg's model was evaluated through changes in explained variance (Δ*R*^2^). Relative importance analyses were performed using the relaimpo package (Grömping, 2007) based on the Lindeman–Merenda–Gold (LMG) method. Statistical significance was set at *p* < 0.05.

The descriptive analyses and data preparation were done in SPSS and Jamovi (The jamovi project, 2024; R Core Team, 2024; Revelle, 2023).

The proportion of missing data was negligible (less than 1% for all study variables). Consequently, listwise deletion was applied before conducting the multivariate analyses.

### 2.5 Ethical considerations

The ethical approval agreement for the present research protocol was approved under ID no. 101, 12/01/2026. The principal investigator of the study was Cădariu Ioana-Eva, affiliated at Tibiscus University from Timisoara.

All participants in this study were adults, currently involved in a romantic relationship. They participated voluntarily. Prior to data collection, participants provided informed consent and were informed about the study aims. The participants were also informed about the anonymity of responses, the right to withdraw at any time without consequences, and the confidential handling of data. No identifying information was collected, and data were stored securely and used exclusively for research purposes in accordance with applicable ethical standards for psychological research.

ChatGPT Plus (GPT-5.5, OpenAI) was used for proofreading and language improvement in selected parts of the manuscript.

## 3 Results

### 3.1 Statistical analyses for content of love messages construct

#### 3.1.1 Exploratory factor analysis

An exploratory factor analysis was conducted (see Table 2) on the 14 items, using maximum likelihood extraction with an Oblimin rotation to examine the instrument's latent structure in Jamovi. Sampling adequacy was excellent (KMO = 0.923; item MSAs = 0.883–0.956), and Bartlett's test of sphericity was significant, χ^2^(91) = 2823, *p* < 0.001, supporting factorability of the correlation matrix. The eigenvalue pattern supported a one-factor solution (Factor 1 eigenvalue = 5.92; next eigenvalue = 0.50). The retained factor explaining 42.3% of the total variance (SS loadings = 5.92). Standardized loadings on the single factor were generally substantial (range = 0.52–0.78 for most items; e.g., CC7 = 0.783, CC2 = 0.765, CC8 = 0.711). Item CC17 showed a weak loading (0.356) and high uniqueness (0.873), which indicated limited shared variance with the common factor. Model fit indices suggested moderate fit for the one-factor model, RMSEA = 0.099, 90% CI [.089, 0.108], TLI =0.856, χ^2^(77) = 410, *p* < 0.001 (BIC = −59.4).

**Table 2:** Factor loadings.

#	Items	Factor	Uniqueness
1	C7	0.781	0.390
2	C2	0.755	0.430
3	C8	0.713	0.491
4	C12	0.704	0.504
5	C15	0.700	0.510
6	C14	0.694	0.519
7	C9	0.666	0.556
8	C3	0.652	0.574
9	C1	0.648	0.580
10	C13	0.605	0.634
11	C6	0.598	0.642
12	C10	0.590	0.652
13	C4	0.529	0.720
14	C17	0.356	0.873

Maximum likelihood extraction with Oblimin rotation.

N = 509.

#### 3.1.2 Confirmatory factor analysis

A confirmatory factor analysis, with 14 variables, was conducted on an independent sample of 325 participants. It was used the weighted least squares mean and variance adjusted (WLSMV) estimator, appropriate for ordinal data (see Table 3). The proposed one-factor model showed an excellent fit to the data, χ^2^(77) = 150.88, CFI = 0.981, TLI = 0.978, RMSEA = 0.054, 90% CI [0.041, 0.067], and SRMR = 0.063. Standardized factor loadings ranged from 0.711 to 0.918.

**Table 3:** Confirmatory factor analysis of the content dimension.

#	Item	Estimate	SE	*z*	*p*	Standardized loading (λ)
	C1	1.000	–	–	–	0.779
	C2	1.035	0.054	19.239	< 0.001	0.806
	C3	1.039	0.057	18.113	< 0.001	0.809
	C4	0.953	0.095	9.995	< 0.001	0.742
	C6	1.163	0.064	18.278	< 0.001	0.906
	C7	1.140	0.076	14.946	< 0.001	0.888
	C8	1.179	0.063	18.624	< 0.001	0.918
	C9	0.913	0.082	11.178	< 0.001	0.711
	C10	1.154	0.060	19.173	< 0.001	0.899
	C12	1.125	0.062	18.062	< 0.001	0.877
	C13	1.115	0.064	17.412	< 0.001	0.868
	C14	1.135	0.068	16.738	< 0.001	0.884
	C15	0.947	0.071	13.351	< 0.001	0.738
	C17	0.982	0.063	15.677	< 0.001	0.765

CFA estimated using WLSMV.

All factor loadings were statistically significant (p < 0.001).

N = 509.

These findings provide strong support for the unidimensional structure of the scale and confirm the factor solution identified in the exploratory analyses.

The better fit of the CFA compared with the EFA reflects the use of an independent validation sample and a theory-driven confirmatory model.

#### 3.1.3 Reliability analysis

The Content scale, with 14 items, showed excellent internal consistency. Cronbach's alpha was α = 0.906 (standardized α = 0.909; 95% CI [0.90, 0.92]) and McDonald's omega was ω = 0.91. Corrected item–total correlations ranged from 0.342 to 0.776. Although C17 showed the lowest corrected item–total correlation, its removal increased Cronbach's alpha only marginally (from 0.906 to 0.910).

#### 3.1.4 Convergent validity

The related constructs showed adequate variability, with Intimacy (*M* = 102.67, SD = 11.26; *N* = 445), Passion (*M* = 99.28, SD = 13.08; *N* = 445), Commitment (*M* = 102.46, SD = 10.77; *N* = 445), and the Content scale (*M* = 39.05, SD = 4.67; *N* = 446). Pearson correlation analyses revealed strong, positive, and statistically significant associations among all variables (all *ps* < 0.001). Specifically, the Content scale was strongly correlated with Intimacy (*r* = 0.750), Passion (*r* = 0.700), and Commitment (*r* = 0.649). The three related constructs were strongly intercorrelated (*r*s = 0.798–0.848). The results indicate that there is strong evidence for the convergent validity of the Content scale (see Table 4).

**Table 4:** Pearson correlations between the scales.

Variables	1	2	3	4
1. Intimacy	–			
2. Passion	0.848^***^	–		
3. Commitment	0.798^***^	0.822^***^	–	
4. Content	0.750^***^	0.700^***^	0.649^***^	–

N = 445 for correlations among intimacy, passion, and commitment.

N = 400 for correlations involving the content scale due to missing data.

Pearson correlation coefficients are reported.

^***^p < 0.001.

#### 3.1.5 Discriminant validity

Discriminant validity was assessed using the Fornell–Larcker criterion. The Content scale demonstrated excellent composite reliability (CR = 0.959), with an AVE of 0.626 and a square root of AVE (√AVE = 0.791). The maximum shared variance was 0.563. The AVE exceeded MSV (see Table 5). The square root of AVE was greater than the correlations between the Content scale and the related constructs: Content with Intimacy (*r* = 0.750), Content with Passion (*r* = 0.700) and with Commitment (*r* = 0.649).

**Table 5:** Construct reliability and validity indices.

Construct	CR	AVE	√AVE	MSV
Content Validity Scale	0.959	0.626	0.791	0.563

These results support acceptable discriminant validity.

#### 3.1.6 Measurement invariance across gender

A multi-group confirmatory factor analysis (MG-CFA) was conducted to examine whether the Content scale was measured equivalently across women and men in R. Configural, metric, scalar, and strict invariance models were tested using the WLSMV estimator. The configural model showed a good fit to the data (CFI = 0.985, TLI = 0.983, RMSEA = 0.058, SRMR = 0.082). Constraining factor loadings, thresholds, and residual variances across gender resulted in negligible changes in model fit (all |ΔCFI| ≤ 0.004 and all |ΔRMSEA| ≤ 0.008). These findings support configural, metric, scalar, and strict measurement invariance, indicating that the Content scale functions equivalently across women and men (see Table 6).

**Table 6:** Measurement invariance of the content scale across gender.

Model	CFI	TLI	RMSEA	SRMR	ΔCFI	ΔRMSEA	Decision
Configural	0.985	0.983	0.058	0.082	—	—	Supported
Metric	0.988	0.987	0.050	0.092	0.003	−0.008	Supported
Scalar	0.984	0.983	0.057	0.081	−0.004	0.007	Supported
Strict	0.988	0.988	0.048	0.092	0.003	−0.008	Supported

According to the criteria proposed by Chen (2007), measurement invariance was supported, as all changes in CFI (|ΔCFI| ≤ 0.010) and RMSEA (|ΔRMSEA| ≤ 0.015) remained below the recommended cut-off values across all invariance models.

#### 3.1.7 Criterion-related (incremental) validity

Criterion-related validity of the Content scale was assessed using multiple linear regression (enter method). The Content scale was considered a dependent variable, while Intimacy, Passion, and Commitment were entered simultaneously as predictors. The regression model was statistically significant, F(3, 396) = 179.40, *p* < 0.001, explaining 57.6% of the variance in Content scores (*R*^2^ = 0.576, adjusted *R*^2^ = 0.573).

Intimacy was the strongest unique predictor of the Content scale (β = 0.545, *p* < 0.001), followed by Passion (β = 0.203, *p* = 0.003). Commitment did not make a significant unique contribution after controlling for the other predictors (β = 0.038, *p* = 0.540). Collinearity diagnostics indicated acceptable levels of multicollinearity (VIFs = 3.54–4.39; tolerance = 0.23–0.28), suggesting that multicollinearity did not substantially influence the regression estimates (see Tables 7, 8).

**Table 7:** Predictors of content of love messages based on Sternberg's triangular theory dimensions.

Predictor	Estimate	SE	*t*	*p*	Standard estimate	Lower CI (95%)	Upper CI (95%)
Constant	9.274	1.438	6.448	**< 0.001**	–	6.447	12.101
Intimacy	0.210	0.026	7.954	**< 0.001**	0.545	0.411	0.680
Passion	0.069	0.023	2.971	**0.003**	0.203	0.069	0.338
Commitment	0.016	0.025	0.614	**0.540**	0.038	−0.083	0.159

**Table 8:** VIF and tolerance coefficients.

Predictor	VIF	Tolerance
Intimacy	4.39	0.228
Passion	4.38	0.228
Commitment	3.54	0.282

Tolerance, 1/VIF. VIF values below 5 and tolerance values above 0.20 indicate that multicollinearity was not a serious concern.

N = 400.

Content is strongly associated with theoretically related constructs of intimacy and passion, but also captures variance not fully explained by commitment.

### 3.2 Modality of love messages

#### 3.2.1 Exploratory factor analysis

To examine the latent structure of the Modality scale, an exploratory factor analysis was performed using maximum likelihood estimation. The sample demonstrated excellent factorability (KMO = 0.97). Guided by the theoretical assumption of a single underlying construct, a one-factor solution was extracted. All items showed substantial factor loadings (0.780–0.864), and communalities ranged from 0.609 to 0.746. The extracted factor explained 66.90% of the total variance. Fit statistics indicated an acceptable representation of the data (RMSR = 0.03, TLI = 0.95, RMSEA = 0.083, 90% CI [.076, 0.090]), supporting the proposed unidimensional structure of the scale (see Table 9).

**Table 9:** EFA factor coefficients.

Item	Factor loading	Communality (*h*^2^)	Uniqueness (*u*^2^)
M7	0.817	0.667	0.333
M9	0.780	0.0609	0.0391
M11	0.824	0.680	0.320
M12	0.797	0.635	0.365
M13	0.840	0.706	0.294
M15	0.864	0.746	0.254
M16	0.811	0.658	0.342
M18	0.845	0.714	0.286
M20	0.804	0.647	0.353
M23	0.826	0.683	0.317
M24	0.813	0.661	0.339
M29	0.836	0.698	0.302
M31	0.782	0.611	0.389
M34	0.808	0.652	0.348

ML, maximum likelihood extraction.

h^2^, communality.

u^2^, uniqueness (1 – h^2^).

Factor loadings are standardized coefficients.

#### 3.2.2 Confirmatory factor analysis

A confirmatory factor analysis (CFA) was conducted to examine the factorial structure of the 14-item Modality scale using the WLSMV estimator. The one-factor model demonstrated an excellent fit to the data, χ^2^(77) = 97.28, *p* = 0.059, CFI = 0.998, TLI = 0.997, RMSEA = 0.029, 90% CI [0.000, 0.045], and SRMR = 0.028. All standardized factor loadings were statistically significant (*p* < 0.001) and ranged from 0.822 to 0.959, providing strong support for the unidimensional structure of the Modality construct (see Table 10).

**Table 10:** Standardized factor loadings for the confirmatory factor analysis of the modality dimension.

Item	Standardized loading (λ)	*R* ^2^	Uniqueness (1–*R*^2^)
M7	0.953	0.908	0.092
M9	0.866	0.750	0.250
M11	0.950	0.903	0.097
M12	0.896	0.804	0.196
M13	0.959	0.919	0.081
M15	0.909	0.826	0.174
M16	0.903	0.815	0.185
M18	0.937	0.878	0.122
M20	0.913	0.834	0.166
M23	0.943	0.890	0.110
M24	0.893	0.798	0.202
M29	0.885	0.783	0.217
M31	0.822	0.675	0.325
M34	0.934	0.872	0.128

λ, standardized factor loading; R^2^, explained variance; Uniqueness, 1–R^2^.

To further evaluate the proposed measurement structure, an alternative one-factor model, in which all 28 items loaded on a single latent Love factor, was also tested. Although the one-factor model demonstrated excellent fit (χ^2^(350) = 453.99, *p* < 0.001, CFI = 0.991, TLI = 0.991, RMSEA = 0.031, 90% CI [0.022, 0.039], SRMR = 0.051), the hypothesized two-factor correlated model showed a consistently better fit (χ^2^(349) = 428.93, *p* = 0.002, CFI = 0.993, TLI = 0.993, RMSEA = 0.027, 90% CI [0.017, 0.035], SRMR = 0.048), supporting the theoretical distinction, claimed by the LRMT, and the clinical different treatment approach, regarding the Content and Modality realities.

#### 3.2.3 Reliability analysis

The 14-item Modality scale demonstrated excellent internal consistency, with a Cronbach's alpha of 0.964 (95% CI [0.960, 0.967]). The average inter-item correlation was 0.669, indicating a high degree of homogeneity among the items. Corrected item–total correlations ranged from 0.754 to 0.837, exceeding the recommended threshold of 0.30. Cronbach's alpha did not increase following the deletion of any item (α = 0.960–0.962), indicating that all items contributed to the reliability of the scale.

#### 3.2.4 Convergent validity

To examine convergent validity, we used Pearson correlations between the Modality scale and Intimacy, Passion, and Commitment. The Modality scale was strongly and positively correlated with Intimacy (*r* = 0.727, 95% CI [0.680, 0.769], *p* < 0.001). It was strongly correlated with Passion (*r* = 0.668, 95% CI [0.613, 0.717], *p* < 0.001), and moderately correlated with Commitment (*r* = 0.537, 95% CI [0.467, 0.601], *p* < 0.001). The results show good convergent validity. Higher levels of relational modality are associated with greater intimacy, passion, and commitment (see Table 11).

**Table 11:** Pearson correlations between the scales.

Variables	1	2	3
1. Modality			
2. Intimacy	0.727^*^		
3. Passion	0.668^*^	0.848^***^	
4. Commitment	0.537^*^	0.798^***^	0.822^***^

Pearson correlation coefficients.

^*^p < 0.05, ^***^p < 0.001.

*N* = 509.

#### 3.2.5 Discriminant validity

Discriminant validity was assessed using the Fornell–Larcker criterion and the comparison between average variance extracted (AVE) and maximum shared variance (MSV). For Modality AVE was high (0.74) and exceeded the MSV (0.53). Additionally, the square root of AVE (√AVE = 0.86) was greater than the construct's correlations with Intimacy (*r* = 0.73), Passion (*r* = 0.67), and Commitment (*r* = 0.54). This supports adequate evidence of discriminant validity (see Table 12).

**Table 12:** Construct reliability and validity indices.

Construct	CR	AVE	√AVE	MSV
Factor 1	0.97	0.74	0.86	0.53

N = 509.

#### 3.2.6 Measurement invariance across gender

A multi-group confirmatory factor analysis (MG-CFA) was conducted to examine measurement invariance of the Modality scale across gender. The configural model demonstrated an excellent fit to the data (CFI = 0.998, TLI = 0.998, RMSEA = 0.030, SRMR = 0.041). Constraining factor loadings (metric invariance), thresholds (scalar invariance), and residual variances (strict invariance) resulted in negligible changes in model fit (ΔCFI ≤ 0.001; ΔRMSEA ≤ 0.008), supporting configural, metric, scalar, and strict measurement invariance across women and men (see Table 13). These findings indicate that the Modality scale measures the same latent construct equivalently across gender, allowing meaningful comparisons between female and male participants.

**Table 13:** Measurement invariance of the content scale across gender.

Model	CFI	TLI	RMSEA	SRMR	ΔCFI	ΔRMSEA	Decision
Configural	0.998	0.998	0.030	0.041	–	–	Supported
Metric	0.999	0.999	0.023	0.052	+0.001	−0.007	Supported
Scalar	0.998	0.998	0.031	0.041	−0.001	+0.008	Supported
Strict	0.999	0.999	0.023	0.052	+0.001	−0.008	Supported

According to the criteria proposed by Chen (2007), measurement invariance was supported, as all changes in CFI (|ΔCFI| ≤ 0.010) and RMSEA (|ΔRMSEA| ≤ 0.015) remained below the recommended cut-off values across all invariance models.

#### 3.2.7 Criterion-related (incremental) validity

The criterion-related (incremental) validity of the Modality scale was assessed using multiple regression analysis with the enter method. Modality was the dependent variable, whereas Intimacy, Passion, and Commitment were entered together as predictors.

The regression model was statistically significant, F(3, 435) = 180.80, *p* < 0.001, and explained 55.5% of the variance in Modality (*R*^2^ = 0.555, Adjusted *R*^2^ = 0.552). Intimacy was the strongest positive predictor (*B* = 0.023, *t* = 10.30, *p* < 0.001), followed by Passion (*B* = 0.009, *t* = 4.53, *p* < 0.001). Commitment showed a significant negative regression coefficient (*B* = −0.009, *t* = −4.04, *p* < 0.001) after controlling for Intimacy and Passion, suggesting a suppression effect attributable to the substantial overlap among the three dimensions of the Triangular Theory of Love. Multicollinearity diagnostics indicated acceptable levels of collinearity (VIFs = 3.46–4.47) (see Table 14).

**Table 14:** Model coefficients for modality.

Predictor	B	SE	t	p	VIF	Tolerance
Intercept	0.392	0.129	3.05	0.002	—	
Intimacy	0.023	0.002	10.30	< 0.001	4.00	0.250
Passion	0.009	0.002	4.53	< 0.001	4.47	0.224
Commitment	−0.009	0.002	−4.04	< 0.001	3.46	0.289

B, unstandardized regression coefficient; SE, standard error; Tolerance, 1/VIF; VIF, variance inflation factor. Tolerance values greater than 0.20 and VIF values below 5 indicate that multicollinearity was not a serious concern.

*N* = 509.

### 3.3 Predictive and incremental validity of the Marici Love Messages Scale

Hierarchical regression analyses supported the incremental validity of the Love Messages dimensions beyond Sternberg's Triangular Theory of Love. Across four of the five outcomes, both Content and Modality contributed significant additional explained variance after controlling for Intimacy, Passion, and Commitment. The largest increases in explained variance were observed for Negative Perception of Sex Life (Δ*R*^2^ = 0.191) and Communication (Δ*R*^2^ = 0.068) (see Table 15).

**Table 15:** Incremental validity of the proposed scale over Sternberg's dimensions (hierarchical regression).

Dependent Variable	Step	Predictors	*R* ^2^	ΔR^2^	F/ΔF	Significant predictors (β, *p*)
**1. Desire for physical separation**	Step 1	Intimacy, passion, commitment	0.270	—	F(3,356) = 43.79^***^	Intimacy (β = −0.45, *p* < 0.001)
	Step 2	+ Modality	0.316	0.047	ΔF(1,355) = 24.54^***^	Intimacy (β = −0.26, *p* = 0.011), Modality (β = −0.33, *p* < 0.001)
	Step 3	+ Content	0.326	0.010	ΔF(1,354) = 5.32^*^	Intimacy (β = −0.23, *p* = 0.027), Modality (β = −0.19, *p* = 0.036), Content (β = −0.21, *p* = 0.022)
**2. Communication**	Step 1	Intimacy, passion, commitment	0.368	—	F(3,356) = 69.14^***^	Intimacy (β = −0.50, *p* < 0.001), Passion (β = −0.23, *p* = 0.010)
	Step 2	+ Modality	0.421	0.052	ΔF(1,355) = 32.91^***^	Intimacy (β = −0.30, *p* = 0.002), Modality (β = −0.35, *p* < 0.001)
	Step 3	+ Content	0.437	0.016	ΔF(1,354) = 10.33^**^	Intimacy (β = −0.25, *p* = 0.007), Modality (β = −0.17, *p* = 0.039), Content (β = −0.27, *p* = 0.001)
**3. Emotional Infidelity (IE)**	Step 1	Intimacy, passion, commitment	0.109	—	F(3,356) = 14.49^***^	Intimacy (β = −0.36, *p* < 0.001)
	Step 2	+ Modality	0.130	0.021	ΔF(1,355) = 8.68^**^	Intimacy (β = −0.23, *p* = 0.042), Modality (β = −0.22, *p* = 0.004)
	Step 3	+ Content	0.145	0.015	ΔF(1,354) = 6.24^*^	Content (β = −0.25, *p* = 0.013)
**4. Negative Perception of Sex Life (NPOSL)**	Step 1	Intimacy, passion, commitment	0.342	—	F(3,356) = 61.80^***^	Intimacy (β = −0.45, *p* < 0.001), Passion (β = −0.25, *p* = 0.006)
	Step 2	+ Modality	0.499	0.156	ΔF(1,355) = 118.86^***^	Modality (β = −0.60, *p* < 0.001)
	Step 3	+ Content	0.534	0.035	ΔF(1,354) = 26.75^***^	Modality (β = −0.34, *p* < 0.001), Content (β = −0.39, *p* < 0.001)
**5. Hope in the relationship**	Step 1	Intimacy, passion, commitment	0.690	—	F(3,356) = 264.40^***^	Intimacy (β = 0.53, *p* < 0.001), Commitment (β = 0.24, *p* < 0.001)
	Step 2	+ Modality	0.693	0.002	ΔF(1,355) = 2.77, ns	Intimacy (β = 0.49, *p* < 0.001), Commitment (β = 0.26, *p* < 0.001)
	Step 3	+ Content	0.696	0.003	ΔF(1,354) = 3.64, ns	Intimacy (β = 0.47, *p* < 0.001), Commitment (β = 0.24, *p* < 0.001)

Step 1, Intimacy, passion, and commitment.

Step 2, Step 1 + Modality.

Step 3, Step 2 + Content.

^*^p < 0.05, ^**^p < 0.01, ^***^p < 0.001.

*N* = 509.

The results for the relative importance of Sternberg and Marici Love Messages predictors are presented below (see Table 16).

**Table 16:** Relative importance of Sternberg and love messages predictors.

Dependent Variable	Intimacy	Passion	Commitment	Sternberg total	Modality	Content	Love messages total
Desire for physical separation	21.9%	15.7%	10.8%	**48.4%**	25.7%	25.9%	**51.6%**
Communication	22.0%	17.0%	11.1%	**50.1%**	23.8%	26.1%	**49.9%**
Emotional Infidelity (IE)	21.9%	11.6%	10.0%	**42.4%**	22.3%	34.3%	**57.6%**
Negative perception of sex life (NPOSL)	14.3%	12.3%	8.3%	**35.0%**	32.0%	33.1%	**65.0%**
Hope in the relationship	29.7%	20.4%	23.1%	**73.2%**	12.1%	14.7%	**26.8%**

Relative importance percentages were calculated using the LMG (Lindeman, Merenda, & Gold) method.

This approach decomposes the total explained variance (R^2^) of the final regression model and estimates the average contribution of each predictor across all possible orders of entry into the model.

The resulting percentages represent each predictor's relative contribution to the total explained variance and sum to 100%.

*N* = 509.

## 4 Discussions

The present article aimed to examine the psychometric characteristics of the Marici Love Messages Scale, focusing on evaluating the Content and Modality subscales. The measure is based on LRMT, a theory that proposes that love messages are composed of two complementary components: the action (Content) and how it is done (Modality). Both dimensions are integral parts of the same relational process—the transmission and interpretation of love messages.

This conceptualization emerged from over a decade of couple therapy practice, where the distinction between Content and Modality proved clinically meaningful. For example, a partner may acknowledge that her spouse spends time with her, which represents a potentially positive relational behavior (Content). However, if during that time he remains emotionally disengaged, continuously checking his phone or appearing inattentive, the Modality conveys indifference rather than care. Consequently, the overall experience may be perceived as less of a love message and more as a rejection message. In this sense, the relational meaning of an interaction depends not only on what is done, but also on how it is enacted.

The distinction is also clinically useful. In therapeutic settings, practitioners can examine individual Content and Modality indicators, combine questionnaire results with interview data, and identify whether difficulties arise primarily from the absence of loving behaviors, from the manner in which they are expressed, or from both. This facilitates more precise assessment and intervention planning.

At a theoretical level, the relationship between Content and Modality reflects a longstanding philosophical question concerning the inseparability of form and content. Content requires a form through which it can be expressed, while form necessarily conveys some content. Content of love messages refers to actions perceived as an expression of love from the other partner, while modality refers to the manners in which these actions are done, being psychologically and conceptually different. Accordingly, LRMT views Content and Modality not as separate psychological constructs but as distinct conceptual and interventional dimensions of a broader latent construct, namely Love Messages.

### 4.1 Factor structure and reliability

The results provide consistent support for the proposed measurement structure. Both the Content and Modality scales showed clear unidimensional structures and high internal consistency. This indicates that the two LRMT dimensions can be assessed reliably. Although the two factors were strongly correlated, they remained distinguishable, supporting their use as complementary dimensions of love messages in both research and clinical assessment.

Measurement invariance across gender was supported at the configural, metric, scalar, and strict levels, as changes in CFI (ΔCFI ≤ 0.010) and RMSEA (ΔRMSEA ≤ 0.015) met the recommended criteria (Chen, 2007).

### 4.2 Convergent and discriminant validity

The convergent validity findings indicate that both LRMT dimensions are closely related to established components of romantic love, particularly intimacy and passion, while maintaining meaningful associations with commitment. These results support the theoretical assumption that love messages reflect central relational processes rather than unrelated interpersonal behaviors. The discriminant validity analyses showed that both dimensions satisfied the Fornell–Larcker criterion, indicating that they capture variance beyond existing love constructs. Although Content and Modality were strongly associated, this overlap is theoretically expected because both represent complementary aspects of the same relational phenomenon—what partners do to express love and how these actions are conveyed. Together, these findings support the construct validity of the proposed framework while suggesting that the two dimensions assess related but distinguishable aspects of couple functioning.

### 4.3 Predictive and incremental validity

The findings suggest that Content and Modality capture related but distinct aspects of love messages. If Content were redundant with Modality, the addition of Content at Step 3 would not have produced significant improvements in model fit. Instead, the results indicate that both dimensions contribute unique predictive information beyond Sternberg's Intimacy, Passion, and Commitment dimensions, supporting the incremental validity of the proposed framework.

Relative importance analyses further supported the predictive validity of the scale. For Desire for Physical Separation, Communication, Emotional Infidelity, and Negative Perception of Sex Life, the combined contribution of Content and Modality exceeded or closely matched that of Sternberg's dimensions, indicating that the proposed Love Messages dimensions explained substantial unique variance beyond the traditional components of love. In contrast, Hope in the Relationship was primarily explained by Intimacy and Commitment, with the Love Messages dimensions providing only minimal additional explanatory value. These findings suggest that the Love Messages dimensions capture unique aspects of relationship functioning that are not fully represented by existing love constructs.

## 5 Conclusions

The purpose of the present article was to explore the psychometric properties of Marici Love Messages measure.

There are some limitations of the present study. Firstly, the study relied on self-report measures, which may be affected by social desirability and subjective bias. Secondly, the cross-sectional design prevents conclusions about causal relationships or changes in love dynamics over time. Thirdly, the sample was drawn from a single cultural and linguistic context, which may limit the generalizability of the findings to other populations, beyond Romanian culture.

The main contribution of this measure is the introduction of two new dimensions of romantic love that have are new to the scientific literature. Based on the Love and Rejection Messages Theory (LRMT), it offers a different way of understanding and measuring love by focusing on the exchange of messages of love deconstructed in content and modality. In addition, the scale shows that it can measure romantic love even better in relation to other relevant variables. Practically, this measure can measure whether love is deficient in its content or modality as a whole. However, in clinical practice it would be advisable to inspect each item and find low score items as a reflection of a deficient or poor flux of love messages between partners. The scale represents one step forward in developing a practical way to develop the theory and its derivatives. As a matter of fact, the study shows that the Content and Modality scales predict essential relationship outcomes, beyond Sternberg's Triangular Theory of Love. This clearly indicates that the new instrument brings to the table a unique explanatory value, rather than measuring constructs that are already covered by existing scales.

Regarding future research, longitudinal studies are needed to examine how content and modality evolve over time and predict relationship stability or change. The scale should be validated in diverse cultural and linguistic contexts to test cross-cultural invariance. Future research should examine dyadic processes by collecting data from both partners simultaneously to analyze agreement, discrepancy, and reciprocal influences between partners' love messages, as the present study collected data only from one dyadic partner. In addition, as this is the first try to validate a measure, new instruments can be developed based on the LRMT.

## Data Availability

The datasets presented in this study can be found in online repositories. The names of the repository/repositories and accession number(s) can be found below: https://osf.io/u6z9w/.
