# Supplementary material for: Psychometric evaluation of a new love scale, measuring the content and modality dimensions, based on love and rejection messages theory
Source: Front Psychol. 2026 Aug 14;17:1806481. doi: 10.3389/fpsyg.2026.1806481 (PMC13522160; doi:10.3389/fpsyg.2026.1806481)
Supplement: Supplementary file 1 [file Data_Sheet_1.pdf]

Annexes

Scale: Marici Love Messages Scale (MLMS) in Romanian and English

Subscale 1: Content of Love Messages (CLM)

The following questionnaire focuses on the activities your partner does with you (content) in order to make you happy in couple. Please read each activity listed and indicate the degree you partner did or participated in these activities, in the last 6 months, as part of couple life.

1 (absent sau puțin/absent or little)
2 (insuficient/insufficient)
3 (suficient sau mai mult/enough or much more)

Activitate/Activity
C1. Petrece timp cu mine.<br>Spends time with me.
C2. Mă ascultă când îi vorbesc.<br>Listens to me when I talk to her/him.
C3. Îmi face complimente.<br>Compliments me.
C4. Se comportă firesc când mă întâlnesc cu prietenii mei.<br>Behaves naturally when I meet with my friends.
C6. Mă întreabă ce îmi doresc.<br>Asks me what I want.
C7. Îmi oferă ajutor în activitățile mele personale.<br>Offers to help me with my personal activities.
C8. Discută cu mine.<br>Talks with me.
C9. Doarme lângă mine în pat.<br>Sleeps next to me in bed.
C10. Îmi zice că mă iubește.<br>Tells me that he/she loves me.
C12. Îmi oferă lucruri.<br>Gives me things.
C13. Îmi cere părerea în decizii.<br>Asks for my opinion when making decisions.
C14. Acceptă deciziile mele personale.<br>Accepts my personal decisions.
C15. Întreține relații sexuale cu mine.<br>Has sex with me.
C17. Petrece timp cu membrii familiei mele.

Spends time with members of my family.

Note: © 2026 Marius Marici. All rights reserved.

Subscale 2: Modality of Love Messages (MLM)

The following questionnaire focuses on the way your partner does activities (modality) in the relationship with you as a lover. Please read each activity listed and indicate whether your partner makes you feel like that, in the last 6 months, as part of couple life.

1 (absent sau puțin/absent or little)
2 (insuficient/insufficient)
3 (suficient sau mai mult/enough or much more)

**Item: Modalitate / Item: Modality**
M7. pune relația noastră pe primul loc, comparativ cu alte relații.<br>*puts our relationship first, ahead of other relationships.*
M9. își amintește detalii semnificative despre mine.<br>*remembers important details about me.*
M11. ține cont de ce îmi place, când face ceva pentru mine.<br>*takes into account what I like when doing something for me.*
M12. intuiește ce îmi doresc, fără să mă întrebe.<br>*knows what I want without having to ask me.*
M13. îmi acordă atenție și nu se plictisește de mine.<br>*gives me attention and does not get bored with me.*
M15. mă face să mă simt ca fiind centrul universului său.<br>*makes me feel like I am the center of their universe.*
M16. este primul care îmi acordă atenție atunci când reușesc.<br>*is the first to pay attention to me when I succeed.*
M18. îmi acordă constant atenție.<br>*gives me consistent attention.*
M20. răspunde rapid nevoilor mele, fără amânare.<br>*responds promptly to my needs without delay.*
M23. îmi arată iubirea spontan și nu calculat.<br>shows me love spontaneously rather than in a calculated way.
M24. mesajele de iubire vin din propria sa inițiativă și nu din presiuni externe.<br>*expresses love on his/her own initiative rather than because of external pressure.*
M29. răspunde cu înțelegere și nu îl deranjează ceea ce spun.<br>*responds with understanding and is not bothered by what I say.*
M31. știe cum să mă influențeze, fără să fie insistent sau cerșetor.<br>*knows how to influence me without being pushy or needy.*
M34. este romantic cu mine și nu doar realist.<br>*is romantic with me rather than being only realistic.*

Note: © 2026 Marius Marici. All rights reserved.
